# Emotionality of Colors: An Implicit Link between Red and Dominance

**DOI:** 10.3389/fpsyg.2017.00317

**Published:** 2017-03-06

**Authors:** Stijn V. Mentzel, Linda Schücker, Norbert Hagemann, Bernd Strauss

**Affiliations:** ^1^Institute of Sport and Exercise Sciences, University of MünsterMünster, Germany; ^2^Institute of Sport and Exercise Sciences, University of KasselKassel, Germany

**Keywords:** color, Stroop task, red, dominance, emotionality

## Abstract

The color red has been shown to alter emotions, physiology, psychology, and behavior. Research has suggested that these alterations could possibly be due to a link between red and perceived dominance. In this study we examined if the color red is implicitly associated to the concept of dominance. In addition, we similarly hypothesized that blue is implicitly linked to rest. A modified Stroop word evaluation task was used in which 30 participants (23.07 ± 4.42 years) were asked to classify words shown in either red, blue, or gray (control condition), as being either dominant- or rest-related. The responses were recorded and analyzed for latency time and accuracy. The results revealed a significant word type × color interaction effect for both latency times, *F*(2,56) = 5.09, *p* = 0.009, $\eta_{p}^{2}$ = 0.15, and accuracy, *F*(1.614,45.193) = 8.57, *p* = 0.001, $\eta_{p}^{2}$ = 0.23. On average participants showed significantly shorter latency times and made less errors when categorizing dominance words shown in red, compared to blue and gray. The measured effects show strong evidence for an implicit red-dominance association and a partial red-rest disassociation. It is discussed that this association can possibly affect emotionality, with the presentation of red eliciting a dominant emotional and behavioral response.

## Introduction

Colors are omnipresent in everyday life and have been shown to have an expansive and diverse effect on both humans and animals (Elliot and Maier, 2014 for a review; Pryke et al., 2007; Khan et al., 2011). The effects of color range from alterations in emotion (Kaya and Epps, 2004), behavior (Cuthill et al., 1997; Pryke, 2009), and physiology (Dreiskaemper et al., 2013). It has been proposed that different colors are associated with specific meanings. For example, red has been shown to enhance, among others, perceived attractiveness, dominance, anger, and aggressiveness (Elliot and Niesta, 2008; Stephen et al., 2012; Elliot and Maier, 2014; Wiedemann et al., 2015). In addition, a copious amount of studies have investigated the effects of blue, green, and gray (Fetterman et al., 2012; Lichtenfeld et al., 2012; Wiedemann et al., 2015). Green is often selected as this is the opposing color to red in most color-models and has been linked to cognitive restorative effects (Berman et al., 2008), creativity (Lichtenfeld et al., 2012), and safety (Pravossoudovitch et al., 2014). Gray is an optimal achromatic contrast to red and can be matched for chromatic color and lightness. In addition, blue has been linked to comfort, quietness, and restfulness (Kaya and Epps, 2004).

As far back as the research by Adams and Osgold (1973) color has been linked to emotions and emotionality (i.e., the observable behavioral and physiological component of emotion). In their study, they found that light colors are viewed as “good” and dark colors as “bad” by the respondents. As of late, research has focused more on the implicit associations between colors and emotional states (Little and Hill, 2007). Such implicit associations have been found between red and anger (Fetterman et al., 2012), red and failure, and general negative words (Moller et al., 2009), and red and potency (Soriano and Valenzuela, 2009). Most recently, Pravossoudovitch et al. (2014) observed an implicit association between red and danger. In this study, participants were asked to classify words as being either safety- or danger-related, using a variant of the Stroop word evaluation task. Their results showed that words and symbols associated with danger were classified faster when shown in red, compared to green and gray. Overall, these findings indicate that specific words and symbols might trigger changes in emotion and emotionality. For example, red may cause an emotional change (e.g., fear), which in turn could lead to a fight or flight behavioral or physiological response. This type of response to red has already been shown in rhesus monkeys (Khan et al., 2011).

These findings are often discussed within the larger nature versus nurture context (Elliot and Maier, 2014). On one side, specific research focuses on the developmental component, by examining language and color associations (Little and Hill, 2007; Fetterman et al., 2012; Pravossoudovitch et al., 2014). On the other side, there is research with respect to genetic components, by investigating animal behavioral and physiological changes (Pryke et al., 2007; Khan et al., 2011). To explore these proposed effects in humans, Hill and Barton (2005) showed that athletes in red won on average more matches compared to athletes in blue for combat sports. To explain this phenomenon, they hypothesized that the “presence and intensity of red coloration correlates with male dominance and testosterone levels” (p. 293). To assess this statement, Briki and Hue (2016) examined the link between red and dominance. In their study participants were asked to rate a color depicted on a computer screen (either red, blue, or green) on three different scales: dominance, arousal, and pleasure. Their results showed a strong association of red with dominance and arousal, an association between green and arousal, and that blue and green were rated as being more pleasurable compared to red. This study did, however, not clarify whether this red-dominance association was implicit or explicit (e.g., if the participants postulated that red should be scored higher on dominance) and used a very straightforward methodology. However, as the main color theory, the color-in-context theory from Elliot and Maier (2012), suggests that the influence of colors depends on the context of the environment, using a different context and methodical approach to confirm this association is needed in order to confirm and generalize the red-dominance association.

The goal of this study was to verify the presence of this proposed relationship between red and dominance using a Stroop word evaluation task. In addition to red, blue was used to examine the proposed association between blue and rest (Kaya and Epps, 2004), as red and blue are often used as opposing colors in combat sports (e.g., Taekwondo, boxing, wrestling) where perception of dominance plays a role (Hill and Barton, 2005; Hagemann et al., 2008; Dreiskaemper et al., 2013), and finally gray was added as a contrast and control color. This methodology allows us to examine whether the aforementioned association is also present in a different context (word association task). Furthermore, this study will help clarify if the suggested associations are also present when tested in an implicit manner, compared to the previously used methodology. We suspect that words relating to dominance will be classified faster and with less error when shown in red compared to gray and blue. In addition, we hypothesized the same trend for rest-related words depicted in blue.

## Materials and Methods

This study consisted of a pilot and main experiment. The pilot study was used to find the appropriate stimuli (words) for the main experiment, for which participants rated German lexical words as being dominance- and rest-related (see Supplementary Material for further information).

For the main study a modified Stroop task was used, as this method is often implemented to assess implicit associations. The Stroop task makes it possible to examine latency times and errors for pre-programmed stimuli without conscious cognitive processing (Moller et al., 2009; Fetterman et al., 2012; Pravossoudovitch et al., 2014). The study design was approved by the university’s ethics committee. We report how we determined our sample size, all data exclusions (if any), all manipulations, and all measures in the study.

### Participants

The sample size was set *a priori* at 30 participants, based on a target power of 0.80 with medium effect sizes (at minimum), based on previous research (Pravossoudovitch et al., 2014). Thirty university students participated (15 female, mean age = 23.07 years, *SD* = 4.42 years), of which 29 were eventually analyzed. Exclusion criteria were language impairments (1 female with Russian as her mother tongue was therefore excluded), (uncorrected) visual impairments or any type of self-reported color blindness. Participants were allowed to take part in both the pilot study and the main experiment, because we did not assume an interference here. This group (*N* = 14) was first analyzed separately, but did not differ for average latency times or errors made from the group that did not participate in the pilot study, *t*(27) = 0.24, *p* = 0.81, *d* = 0.09 and *t*(27) = 0.10, *p* = 0.92, *d* = 0.03, respectively.

### Design and Procedure

The study was performed on a white computer screen, which was color calibrated before each measurement using a Spider 3 Elite (Datacolor) display calibration tool. This was done to guarantee that the colors displayed on the screen were identical to the colors selected beforehand. The properties of the selected colors were: blue [Lightness Chroma hue (LCh): 53.0, 95.0, 240.0], gray (LCh: 53.0, 0.0, 180.0), and red (LCh: 53.0, 95.0, 40.0, within 2.0 units; Stokes et al., 1992). To further ensure that each participant saw the colors in an indistinguishable fashion, all environmental light sources were controlled using blinds, also the chair height was adjusted to ensure that all participants had the same viewing angle of the screen.

Participants were shown 10 German words presented in either blue, red, or gray and were asked to press a labeled key (left or right arrow) to indicate if a word was dominance- or rest-related. The label position was counterbalanced between left and right across participants.

The main experiment consisted of 180 trials divided into three blocks of 60 trials each. For each block the stimuli were depicted in a randomized order using the randomize function of the software program Inquisit v. 4 (Millisecond Software). This function regulates that each of the six possible combinations of word type (dominance or rest) and color (red, blue, or gray) is shown 10 times per block. This randomization was used in order to combat a potential learning or block effect. Before each trial a fixation cross appeared for 500 ms, then the stimuli was displayed until a response was given or 5000 ms passed. The intertrial interval was 1000 ms. Five practice trials were used for the participants to get accustomed to the assignment of the keys and the general procedure. Word type, color, categorization, and latency were all recorded for each trial using Inquisit. The practice trials were excluded from the statistical analysis. In addition, the wrongfully classified responses (e.g., errors, 4.52% of all responses) were excluded from the latency analysis.

### Statistical Analysis

The remaining trials were classified into six groups and analyzed for possible color, word type, and interaction effects on latency time; the same was inspected for error trials. For this a 2 (word type: dominance or rest) × 3 (color: red vs. blue vs. gray) within-within design ANOVA was performed using SPSS Statistics (version 23, SPSS Inc., Chicago, IL, USA). If necessary, the degrees of freedom were corrected based on Mauchly’s Test of Sphericity using either the Greenhouse-Geisser or Huynh-Feldt estimates of sphericity as proposed in Field (2013).

Significant interaction effects were examined more in depth using a *post hoc* planned contrast test, with α-level being adjusted based on the Bonferroni correction for multiple tests. In addition, preliminary analyses treating gender and laterality as a factor were performed.

## Results

### Latency Times

Primary analysis revealed no main effects for color *F*(1.46,28) = 0.68, *p* = 0.47, $\eta_{p}^{2}$ = 0.02 (Huynh-Feldt adjustment), or word type *F*(1,28) = 2.02, *p* = 0.17, $\eta_{p}^{2}$ = 0.07. There was, however, a significant word type × color interaction effect *F*(2,56) = 5.09, *p* = 0.009, $\eta_{p}^{2}$ = 0.15.

**FIGURE 1:**
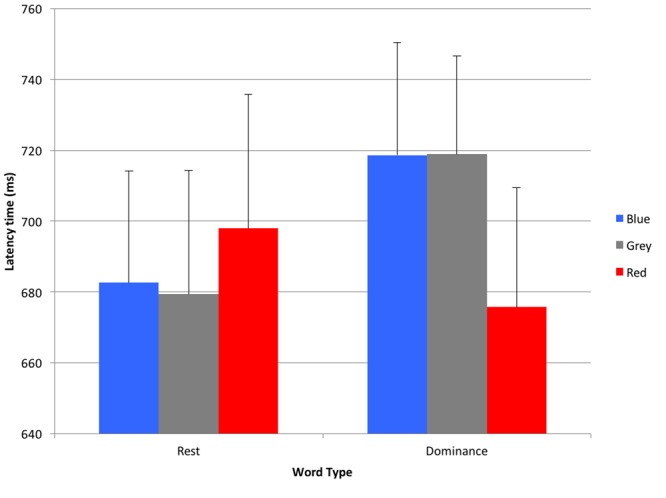
**Latency times for rest and dominance words shown in blue, gray, or red (error bars indicate 1 standard error of each respective mean).** A lower latency time indicates a faster response.

The details of the interaction effect are depicted in **Figure 1**. The *post hoc* planned contrast test revealed that participants showed on average significantly shorter latency times when categorizing dominance words depicted in red (*M* = 675.74 ms, *SD* = 181.34), compared to blue (*M* = 718.57 ms, *SD* = 171.07), *F*(1,28) = 5.98, *p* = 0.021, $\eta_{p}^{2}$ = 0.18, and gray (*M* = 718.87 ms, *SD* = 149.12), *F*(1,28) = 10.61, *p* = 0.003, $\eta_{p}^{2}$ = 0.28. However, the latency did not differ significantly for rest words, with no difference when rest words were shown in blue (*M* = 682.57 ms, *SD* = 169.95), compared to red (*M* = 697.94 ms, *SD* = 204.11), *F*(1,28) = 0.45, *p* = 0.51, $\eta_{p}^{2}$ = 0.02, or gray (*M* = 679.35 ms, *SD* = 188.41), *F*(1,28) = 0.03, *p* = 0.86, $\eta_{p}^{2}$ = 0.001.

Finally, additional analysis showed no gender or laterality effects.

### Errors

**FIGURE 2:**
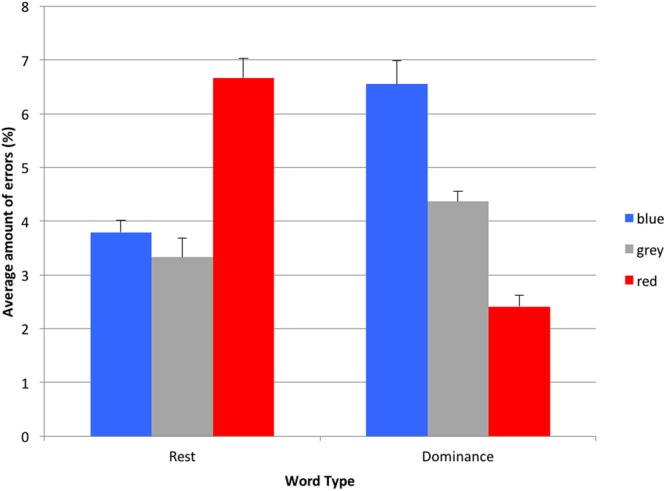
**Average amount of errors for dominance or rest words shown in red, blue, or gray (error bars indicate 1 standard error of each respective mean)**.

On average, only few errors in classification were made during the task (4.52% of all responses), which indicates that the selected words adequately fit to the overlapping categories of dominance and rest. The 2 (word type: dominance or rest) × 3 (color: red vs. blue vs. gray) within-within design ANOVA revealed no main effects for color *F*(2,28) = 1.664, *p* = 0.20, $\eta_{p}^{2}$ = 0.06, or for word type *F*(1,28) = 0.04, *p* = 0.85, $\eta_{p}^{2}$ = 0.001. However, a significant word type × color interaction effect was found *F*(1.614,45.193) = 8.57, *p* = 0.001, $\eta_{p}^{2}$ = 0.23 (Huynh-Feldt adjustment), as can be seen in **Figure 2**.

The *post hoc* planned contrast test analysis revealed that participants made on average significantly less errors when categorizing dominance words depicted in red (*M* = 2.41%, *SD* = 3.66), compared to blue (*M* = 6.56%, *SD* = 7.94), *F*(1,28) = 7.14, *p* = 0.012, $\eta_{p}^{2}$ = 0.20, and gray (*M* = 4.37%, *SD* = 3.46), *F*(1,28) = 7.97, *p* = 0.009, $\eta_{p}^{2}$ = 0.22. Subsequently, more errors were made when rest words were shown in red (*M* = 6.67%, *SD* = 6.67), compared to blue (*M* = 3.79%, *SD* = 4.15), *F*(1,28) = 7.60, *p* = 0.01, $\eta_{p}^{2}$ = 0.21, or gray (*M* = 3.33%, *SD* = 6.36), *F*(1,28) = 12.30, *p* = 0.002, $\eta_{p}^{2}$ = 0.31, with no significant difference in errors between gray and blue, *F*(1,28) = 0.30, *p* = 0.59, $\eta_{p}^{2}$ = 0.01.

Lastly, additional analysis showed no gender or laterality effects.

## Discussion

The findings from this study indicate the existence of an implicit red-dominance association, as was suggested in previous studies (Hill and Barton, 2005; Briki and Hue, 2016). Participants showed on average shorter latency times and made less errors when classifying dominance words depicted in red. These results do not only confirm that the red-dominance association exists, but that this association is also implicit and takes place outside of cognitive processing. The fact that this study showed the presence on an implicit red-dominance association in a different context and using a different methodological approach than Briki and Hue (2016), confirms the strength of this association with regard to the color-in-context theory (Elliot and Maier, 2012).

The results did not verify our secondary hypothesis, namely the presence of an implicit blue-rest association as proposed by Kaya and Epps (2004) as there was no significant difference in latency time and errors made between words shown in gray or blue. This could be possible due to the fact that gray is more associated with rest than with dominance, though this should be examined more extensively in the future. On the other hand, some evidence was found for a red-rest disassociation, as participants made more errors when classifying rest words shown in red. Although, this effect was not found for latency times. These findings suggest that the color red has a much more pronounced effect on latency times and errors than the color blue (with gray being an achromatic control condition) as has been shown in previous studies (Sorokowski and Szmajke, 2011; Briki and Hue, 2016).

Additionally, in our experiment we used a strict measurement design to ensure that all the effects measured were solely due to color effects. This included controlling for environmental light, screen calibration, and the angle at which the screen was perceived (e.g., seat height and distance to screen), which has been shown to influence color perception (Bloj et al., 1999) as well as stern stimuli selection criteria (equal average word length and exclusion of emotionally loaded words). Until now, most computer-based colors studies did not report controlling for all of these external factors (e.g., Fetterman et al., 2012; Pravossoudovitch et al., 2014; Krenn, 2015; Briki and Hue, 2016). Refraining from this type of scrupulous methodology may lead to participants observing slight variations of the preselected colors, which could influence subsequent measurements (Moller et al., 2009). A critical discussion point regarding the design is that we applied a randomize function and did not pre-randomize all 180 trials, which could lead to the occurrence of stimuli blocks. This was combated by having three blocks of 60 trials each to reduce any possible learning or block effects. This type of function has been applied before in other studies using the Stroop Task (Wurm et al., 2004; Krompinger and Simons, 2011).

The implicit association found in this study adds to the existing material and shows that red can have an implicit relationship to certain types of words (Fetterman et al., 2012; Pravossoudovitch et al., 2014). As language is learned and developed over the forming years, these findings strengthen the nurture component of color associations, as has been suggested in previous research (Little and Hill, 2007). This implicit red-dominance association could prompt modulations in emotionality, leading to both a dominant behavioral and physiological response (Hill and Barton, 2005), as has been found in animals (Cuthill et al., 1997; Pryke et al., 2007; Pryke, 2009). For example, previous studies have shown that Gouldian finches with a red color morph demonstrated more dominant behavior (won more contests) and had altered hormonal levels (increase in testosterone and corticosterone) when paired together with black or blue finches (Pryke et al., 2007; Pryke, 2009). Similarly, seeing red activates a dominance concept in humans, which could increase dominance-related behavior, such as engaging in more tackles in soccer or ice-hockey and more dominant-like postures (upright and broad poses). Future research should investigate if seeing red indeed leads to an increase in dominant behavior, as well as examining posture, hormonal levels, etc., which could have compelling practical implications.

## Conclusion

This study showed the presence of an implicit red-dominance association and red-rest disassociation, yielding strong evidence for a link between colors and meanings.

## Ethics Statement

This study was carried out in accordance with the recommendations of the University of Münster ethics guidelines and the ethics committee of the Sport Psychology and Sport Science faculty of the University of Münster with written informed consent from all subjects. All subjects gave written informed consent in accordance with the Declaration of Helsinki. The protocol was approved by the ethics committee of the Sport Psychology and Sport Science faculty of the University of Münster.

## Author Contributions

SM: Data collection and analysis and writing first draft for article. LS: Design of study, supervision, planning, and feedback on written article. NH: Design of study, supervision, planning, and feedback on written article. BS: Design of study, supervision, planning, and feedback on written article. All authors given consent for final version of article.

## Conflict of Interest Statement

The authors declare that the research was conducted in the absence of any commercial or financial relationships that could be construed as a potential conflict of interest.
