# Supplementary material for: Emotionality of Colors: An Implicit Link between Red and Dominance
Source: Front Psychol. 2017 Mar 6;8:317. doi: 10.3389/fpsyg.2017.00317 (PMC5337749; doi:10.3389/fpsyg.2017.00317)
Supplement: Supplementary file 1 [file Table_1.DOCX]

**Supplementary Material**

*Pilot and stimuli*

For the pilot study forty-three university students, all native German speakers, were asked to rate twenty-seven German lexical words, selected from the Berlin Affective Word List Reloaded (BAWL-R), to which degree the words were dominance (e.g. German: Dominanz), force (e.g. German: Kraft) or rest (e.g. German: Ruhe) related using a 6-point scale (1 – not at all, 6 – fits completely). These categories were selected based on previous research(Briki & Hue, 2016; Dreiskaemper et al., 2013; Pravossoudovitch et al., 2014). The participants were also asked to rate the words as being either positive or negative on a similar scale (1 – completely positive, 6 – completely negative), as research has indicated that red was positively associated with negative words and negatively associated with positive words (Moller et al., 2009).
 Based on the results, ten adjectives were selected as stimuli for the main experiment, five dominance-related and five rest-related words (see table 1 for an overview). The five dominance words (might, pressure, active, action and strength) and five rest words (recover, quiet, mild, break and rest) were of equal word length (*M* = 5.4 letters for each). The dominance words were rated as more dominance related (*M* = 4.65, *SD* = 0.57) than the rest words (*M* = 2.43, *SD* = 0.30), *t* (4) = 7.30, *p* = .002, *d* = 3.26. Whereas the rest words were rated as more rest related (*M* = 5.34, *SD* = 0.37) than the dominance words (*M* = 2.72, *SD* = 0.70), *t* (4) = 8.20, *p* = .001, *d* = 3.67.

*Table 1. Overview of selected adjectives in original German, translated English and overall category.*

| **German word** | **English word** | **Category** |
| --- | --- | --- |
| Macht | Power | Dominance |
| Druck | Pressure | Dominance |
| Aktiv | Active | Dominance |
| Aktion | Action | Dominance |
| Stärke | Strength | Dominance |
| Erholung | Recovery | Rest |
| Stille | Calmness | Rest |
| Mild | Mild | Rest |
| Pause | Break | Rest |
| Rast | Rest | Rest |
